# Case report: Pulse cyclophosphamide for treatment of multi-agent-refractory hepatic graft-versus-host disease

**DOI:** 10.3389/fonc.2024.1329893

**Published:** 2024-02-12

**Authors:** Yijun Cai, Amir Ali, Elan Filler, Rua Bayati, Tanjia Toma, Omar Zaki, George Yaghmour, Abdullah Ladha, Karrune Woan, Eric Tam, Preet M. Chaudhary

**Affiliations:** ^1^ Titus Family Department of Clinical Pharmacy, Alfred E. Mann School of Pharmacy and Pharmaceutical Sciences, University of Southern California, Los Angeles, CA, United States; ^2^ Jane Anne Nohl Division of Hematology, Keck School of Medicine, University of Southern California, Los Angeles, CA, United States

**Keywords:** graft-versus-host disease, steroid-refractory, hepatic variant, allogeneic hematopoietic stem cell transplantation, ruxolitinib, pulse cyclophosphamide, case report

## Abstract

Graft-versus-host disease (GVHD) is a common complication in patients receiving allogeneic hematopoietic stem cell transplantation (HSCT). GVHD is characterized as either acute or chronic based on symptomatology and histopathological findings. Despite advancements in disease-targeting therapeutics, steroid-refractory GVHD remains a significant contributor to mortality in HSCT recipients, highlighting the gaps in our understanding of its pathophysiology and treatment strategies. We present the case of a 46-year-old woman diagnosed with acute undifferentiated leukemia, who exhibited persistently elevated levels of serum total bilirubin (T.Bili), alkaline phosphatase (ALP), and liver function tests (LFTs) beginning on [day +201] post-haploidentical peripheral blood stem cell (PBSC) transplantation. The patient received fludarabine/total body irradiation (Flu/TBI) as a myeloablative conditioning regimen and post-transplant cyclophosphamide/tacrolimus/mycophenolate mofetil (PTCy/Tac/MMF) as GVHD prophylaxis. A liver biopsy confirmed the diagnosis of GVHD, while other possible etiologies were excluded by corresponding tests. Initial treatment with prednisone and tacrolimus, and the later addition of ruxolitinib, all showed poor response indicated by worsening T.Bili, ALP, and LFTs at the same time. Based on a multidisciplinary comprehensive assessment, we decided to administer 1,000 mg/m^2^ (1,600 mg) of cyclophosphamide (“pulse Cy”), which resulted in a dramatic improvement in T.Bili and transaminases starting from the very next day. A durable response to pulse cyclophosphamide was observed, as all indicators normalized (“complete response”) within 55 days without relapses. The patient remains in good health with no recurrence of hepatic GVHD. To our knowledge, this is the first case in which Grade IV hepatic GVHD, refractory to multiple agents including steroids, tacrolimus, and ruxolitinib, demonstrated a complete response to pulse cyclophosphamide. The success highlights the potential therapeutic role of cyclophosphamide, a potent and cost-effective chemotherapy agent, in treating multi-agent-refractory GVHD. Large-scale clinical trials are warranted to validate its efficacy in this setting.

## 1 Introduction

Graft-versus-host disease (GVHD) is a significant complication that can occur post-allogeneic hematopoietic stem cell transplantation (HSCT). GVHD results when donor immune cells recognize the recipient’s body as foreign, subsequently triggering an inflammatory reaction (1). GVHD has historically been classified as either acute or chronic, with acute GVHD (aGVHD) conventionally believed to arise within the first 100 days post-transplant, and chronic GVHD (cGVHD) appearing beyond this period. However, current understanding acknowledges the occurrence of late-onset aGVHD, which can materialize beyond the 100-day mark, introducing a potential overlap (“overlap syndrome”) with cGVHD manifestations (2).

cGVHD stands as the primary non-relapse mortality (NRM) driver following allogeneic HSCT, necessitating strategic prophylactic and therapeutic regimens (3). Prophylaxis often comprises a sophisticated array of immunosuppressants with disparate mechanisms, including calcineurin inhibitors (CNIs: cyclosporine [CsA], and tacrolimus [Tac]), antimetabolites (mycophenolate mofetil [MMF] and methotrexate [MTX]), and T-cell depletion strategies (anti-thymocyte globulin [ATG] and post-transplant cyclophosphamide [PTCy]), tailored to the nuances of donor-recipient compatibility (4). Despite rigorous prophylactic protocols, an estimated 20%–80% of recipients might still develop aGVHD, potentially setting the stage for subsequent cGVHD onset (5). There has been an observed escalation in GVHD incidence, attributed primarily to amplified utilization of unrelated/HLA-mismatched donors and G-CSF-mobilized peripheral blood progenitor cells (PBPCs), among others (6).

Therapeutic approaches to GVHD, irrespective of subtype, remain limited, underscoring the importance of enrollment in meticulously designed clinical trials. Glucocorticosteroids (topical and systemic) persist as the mainstay for aGVHD and cGVHD therapy. Intensifying or reinitiating the original immunosuppressive regimen is another first-line approach. Nevertheless, the therapeutic landscape for steroid-refractory cases presents challenges. To date, only ruxolitinib has garnered FDA approval for treating both acute and chronic GVHD, while ibrutinib and belumosudil are approved only for chronic cases. Other guideline-referenced alternatives such as alemtuzumab, alpha-1 antitrypsin (AAT), ATG, basiliximab, CNIs, extracorporeal photopheresis (ECP), and etanercept are backed by limited-scale clinical trials or retrospective cohort studies, thereby casting doubt on their effectiveness (4, 7).

Presented herein is an intriguing case of a 46-year-old female patient diagnosed with acute undifferentiated leukemia, who developed Grade IV hepatic GVHD ~300 days post-haploidentical PBSC transplantation. This patient exhibited resistance to high-dose steroids, escalated tacrolimus, and ruxolitinib, as demonstrated by the persistent elevation of serum total bilirubin (T.Bili), alkaline phosphatase (ALP), and liver function tests (LFTs). The novelty of this case lies in the successful administration of pulse cyclophosphamide—a conventional chemotherapy agent—yielding a substantial reduction in T.Bili, ALP, and the eventual normalization of LFTs, hence shedding light on potential alternative strategies for refractory GVHD management.

## 2 Patient information

### 2.1 Medical history

A 46-year-old female patient with a history of acute undifferentiated leukemia was presented in our report. The diagnosis was associated with myeloproliferative neoplasm (MPN) features; high-risk disease index; non-complexity with trisomy 8; a translocation between chromosomes 8 and X; mutations in *IDH1*, *U2AF1*, *BCOR*, and *GATA1*; and a low level of *CEBPA* (single allele). Notably, a *JAK2* V617F mutation of uncertain clinical significance was identified.

Two years after diagnosis, the patient received high-dose cytarabine (HiDAC) for a bridge to transplant. A bone marrow biopsy before the transplantation showed complete remission (CR3) and minimal residual disease (MRD) negative status by flow. Following the myeloablative conditioning (MAC) regimen consisting of fludarabine and total body irradiation (Flu/TBI: Fludarabine 30 mg/m^2^ on days −4 to −2; total body irradiation, 2 Gy twice per day on days −7 to −5 [12 Gy in total]), she underwent haploidentical PBSC transplantation from her daughter (both CMV positive) on day 0. After the transplant, she was put on a GVHD prophylaxis regimen consisting of PTCy/Tac/MMF (Cyclophosphamide 50 mg/kg on days +3 and +4; Tacrolimus continuous infusion starting at 1 mg/day since day +5, followed by conversion to oral capsules after stable drug level; MMF 1,000 mg orally twice per day since day +5). MMF was discontinued on day +35. Necessary anti-infection prophylaxis (Levofloxacin, isavuconazonium, and letermovir) was administered concurrently.

### 2.2 Clinical findings

Post-transplant, the patient had a stable course except for mild aGVHD manifestations (Grade I) on the skin and mouth until day +201 when T.Bili (1.1 mg/dL), ALP (302 U/L), and LFTs (ALT, 296 U/L; AST, 357 U/L) started to show an uptrend, suggesting possible cGVHD affecting the liver (8). Tacrolimus dosage was increased from 0.5 mg once daily to 1 mg twice daily, and 60 mg of prednisone once daily (~1 mg/kg) was initiated outpatient due to these concerns.

**Figure 1:**
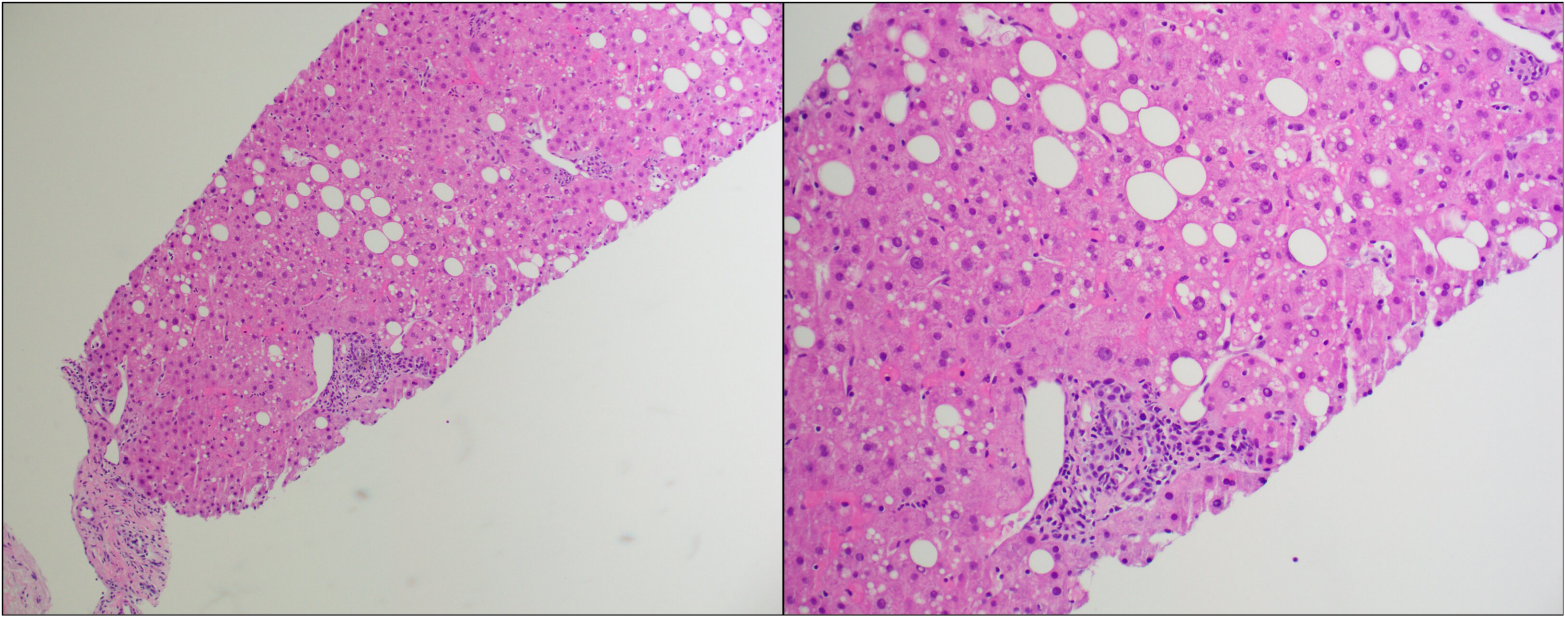
Hematoxylin and eosin (H&E) stain of the first liver biopsy at low power (left) and high power (right).

On day +209, the patient was admitted to USC Norris Cancer Hospital due to the suspicion of steroid-refractory liver cGVHD given continuously increasing levels of T.Bili and transaminases (T.Bili, 2.9 mg/dL; ALP, 287 U/L; ALT, 682 U/L; AST, 595 U/L) concurrent with outpatient steroid treatment. The patient further underwent liver biopsy after admission. The histopathological examination of the biopsy sample revealed features most compatible with GVHD (**Figure 1**). Predominantly lymphocytic inflammation was observed in the portal tracts along with a few neutrophils and eosinophils. A majority of the bile ducts showed inflammatory infiltration and/or damage. In addition to these, the lobules showed mild macrovesicular steatosis and prominent sinusoidal Kupffer cells.

During this hospital stay, despite high steroid dosage (70 mg once daily) and initiation of ruxolitinib (5 mg twice daily) on day +220 (T.Bili, 5.0 mg/dL; ALP, 201 U/L; ALT, 443 U/L; AST, 164 U/L), the patient’s liver GVHD progressed to Grade III liver GVHD in 2 days, with T.Bili of 6.9 mg/dL, ALP of 213 U/L, ALT of 526 U/L, and AST of 234 U/L on day +222, for which a second biopsy was performed (8). The biopsy results once again supported the diagnosis of GVHD with additional findings of steatohepatitis and rare mild pericellular fibrosis. There was prominent cholestasis, minimal portal inflammation, and bile duct damage present. Mild steatosis was noted and some cells showing ballooning degeneration.

The patient was discharged on day +230, with continued outpatient management of prednisone (1 mg/kg/day), tacrolimus (2 mg twice daily), and ruxolitinib (5 mg twice daily). However, she was readmitted to the hospital on day +240 due to sharply uptrending levels of T.Bili (14.6 mg/dL), ALP (262 U/L), and LFTs (ALT, 784 U/L; AST, 212 U/L) and put on 60 mg of prednisone twice daily (~2 mg/kg/day), 3 mg tacrolimus twice daily, and 5 mg of ruxolitinib twice daily. A slight but transient improvement in the lab values (T.Bili, 11.5 mg/dL; ALP, 230 U/L; ALT, 597 U/L; AST, 138 U/L) was observed for a period of 2 days. Nevertheless, this was swiftly followed by a rapid progression to Grade IV liver GVHD on day +245 (T.Bili, 15.5 mg/dL; ALP, 267 U/L; ALT, 649 U/L; AST, 181 U/L) (8). Owing to the hepatic insufficiency, ruxolitinib had to be decreased to 5 mg once daily 4 days after re-hospitalization and discontinued on day +253 (a total of 33 days of use) given her extremely persistently abnormal high levels of T.Bili (>10 × ULN).

### 2.3 Therapeutic intervention

**Figure 2:**
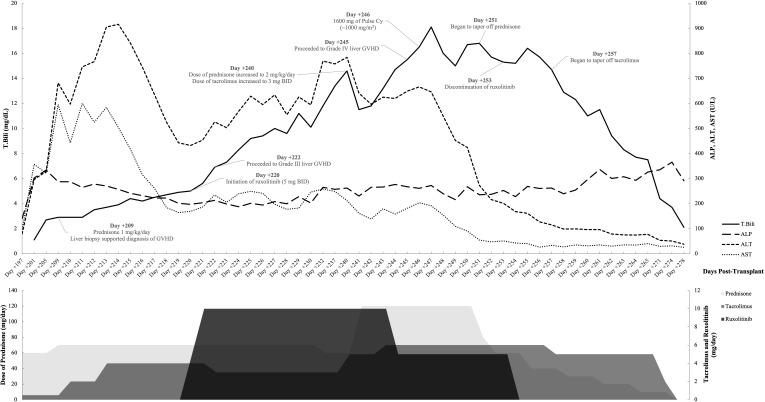
Changes in hepatic panels overtime with concurrent medications.

The potent and enduring therapeutic effect of pulse cyclophosphamide (1,000 mg/m^2^) once administered on day +246) on mitigating steroid-refractory hepatic GVHD was corroborated by the dramatic and sustained improvement in persistent lab indices, beginning on the second day following administration (T.Bili, 16.0 mg/dL; ALP, 239 U/L; ALT, 549 U/L; AST, 154 U/L). The decrement in these lab values was so pronounced that hepatic GVHD reverted to Grade I (T.Bili, 4.4 mg/dL; ALT, 53 U/L; AST, 29 U/L) within a span of 25 days (day +271) post-administration of pulse cyclophosphamide, with few rebounds despite the tapering of steroid therapy (days +251 to +264) and tacrolimus (days +257 to +266). The abnormalities in T.Bili and LFTs were entirely resolved (achieving the “complete response”) by day +301, 55 days after the administration of pulse cyclophosphamide, with a subsequent normalization of ALP values (9). Throughout the treatment and follow-up period, the patient demonstrated good adherence to the prescribed intervention, and there were no significant adverse or unanticipated events reported. Changes in hepatic panels and doses of concurrent medications have been summarized in **Figure 2**.

### 2.4 Follow-up and outcomes

The patient has maintained a sustained remission from hepatic GVHD recurrence since that point. During the most recent clinic visit (2 years post-transplant), comprehensive follow-up assessments were conducted to evaluate the patient’s current health status and response to the treatment. The laboratory results indicated normal liver function (T. Bili, 0.2 mg/dL; ALP, 145 U/L; ALT, 21 U/L; AST, 28 U/L), supporting favorable response to the pulse cyclophosphamide given the severity of the hepatic GVHD at the outset of the intervention.

The patient reported feeling very well and did not express any current health complaints, aligning with the objective clinical findings and suggesting a positive overall impact on the patient’s quality of life post-treatment. Additionally, the bone marrow biopsy (BMBx) conducted during this visit showed no evidence of minimal residual disease (MRD).

## 3 Discussion

### 3.1 Guideline-based management and steroid refractoriness

In managing the case of our patient, established therapeutic guidelines for GVHD were followed, but unfortunately, there is no consensus on treatment options for steroid-refractory GVHD in current guidelines (4). With the initial detection of an upward trend in T.Bili, ALP, and LFTs, indicative of a potential hepatic manifestation of GVHD on [day +201], the regimen of tacrolimus was promptly escalated and systemic corticosteroids initiated, specifically prednisone administered at ~1 mg/kg (later ~2 mg/kg). This immediate intervention resonates with the standard first-line approach as prescribed in numerous guidelines for the management of Grades II–IV GVHD, given the histopathological findings in her liver biopsies. The emphasis lies on the deployment of systemic corticosteroids, barring cases where contraindications or severe intolerance exist.

Despite the utilization of therapeutic strategies recommended in guidelines, the patient’s condition evolved into progression, typically observed in 40%–50% of GVHD cases (5, 10). Her disease state transitioned into steroid-refractory GVHD, a condition unfortunately correlated with high mortality rates (5, 10). This progression was underscored by the observation that the severity of her hepatic GVHD escalated rapidly, moving from Grade II to Grade IV in a compressed timeframe, in spite of intensive steroid therapy and the commencement of tacrolimus and ruxolitinib.

While ruxolitinib is known to have hepatotoxic potential, we strictly followed the recommended dosage guidelines during its administration (4, 11). It is essential to highlight that the deterioration in liver function markers (i.e., T.Bili, ALP, ALT, and AST) began before the introduction of ruxolitinib, indicating an independent progression of hepatic GVHD. Moreover, the initiation of pulse cyclophosphamide treatment overlapped with the ongoing ruxolitinib therapy, and we observed a significant improvement in liver function parameters post-pulse cyclophosphamide even as ruxolitinib continued. This suggests that the amelioration of hepatic symptoms was not directly related to the cessation or reduction in ruxolitinib, which, instead, is more likely attributable to the introduction and effectiveness of pulse cyclophosphamide.

An acknowledged weakness of our case is the lack of trial of ibrutinib and belumosudil (not accessible at the time of this patient’s case, as it just received FDA approval in July 2021), other FDA-approved medications for the treatment of chronic GVHD (cGVHD). This omission might limit the scope of our analysis, particularly in comparing the efficacy and tolerability of different FDA-approved treatments for cGVHD. Future cases could benefit from incorporating a broader range of approved therapeutic agents (i.e., ibrutinib and belumosudil) and other potential choices mentioned in the guideline, to comprehensively assess and optimize treatment strategies for cGVHD.

### 3.2 Exploring other roles of cyclophosphamide in tackling GVHD in addition to PTCy

As a well-known chemotherapeutic agent, cyclophosphamide has historically been deployed in several GVHD management strategies. Early evidence for post-transplant cyclophosphamide (PTCy) came from studies like the one conducted by O’Donnell et al. in 2002, which demonstrated successful engraftment following non-myeloablative hematopoietic stem cell transplantation with cyclophosphamide administered at 50 mg/kg on days +3 and +4 for GVHD prophylaxis, even in cases of HLA-mismatched marrow from first-degree relatives (12).

The prophylaxis regimen that our patient was put on, PTCy/Tac/MMF, has recently gained favor as a standard of care in mismatched transplants due to its superior GVHD prophylaxis effect, as attested by the latest phase III clinical trial (BMT CTN 1703). It was demonstrated that this regimen resulted in a ≥15% higher 1-year GRFS compared to Tac/MTX without an increased risk of relapse or death. This combination regimen, therefore, is positioned to become the standard of care for GVHD prophylaxis even from closely matched donors receiving reduced-intensity conditioning (RIC) (13).

Despite an initial response, our patient developed GVHD, which eventually became refractory to steroids, tacrolimus, and ruxolitinib. As a salvage therapy, pulse cyclophosphamide was administered as a one-time pulse dose. This approach is backed by research indicating that even a high dose of cyclophosphamide up to 7,000 mg/m^2^ does not cause irreversible marrow damage (14). Cyclophosphamide’s unique pharmacological properties result in maximal immunosuppression without myeloablation. The basis for this lies in the differential expression of liver cytosolic aldehyde dehydrogenases (ALDHS) between various cell types. Hematopoietic stem cells express higher levels of ALDHS, which confers cellular resistance to cyclophosphamide, whereas B lymphocytes, T lymphocytes, and natural killer (NK) cells express lower levels, making them extremely sensitive to cyclophosphamide’s cytotoxic properties (15).

In summary, this patient case reflects a new strategic deployment of cyclophosphamide post stem cell transplantation, offering a promising therapeutic strategy for challenging steroid-refractory GVHD cases.

### 3.3 Prior evidence in treating steroid-refractory GVHD with pulse cyclophosphamide

Mayer et al. first introduced the concept of using pulse cyclophosphamide in 15 patients with steroid-resistant GVHD, laying the groundwork for further investigations. Interestingly, this study highlighted a disparity in response rates depending on the involved organs, with intestinal subtypes demonstrating the poorest response. Notably, liver involvement responded exceptionally well, a finding that contradicts previous reports and prompted further investigations into the use of pulse cyclophosphamide specifically for hepatic GVHD (16). Subsequently, they conducted a retrospective analysis focusing on 21 cases of the steroid-refractory hepatitic variant of liver GVHD, further confirming the efficacy of pulse cyclophosphamide. The complete response rate achieved was promising, especially when compared to other studies targeting the same disease variant. The study, however, did not include patients with Grade IV hepatic GVHD, presenting a limitation to the general applicability of the findings (17).

Collectively, both studies by Mayer and colleagues established pulse cyclophosphamide as an effective treatment option for steroid-refractory GVHD, noting its rapid onset and manageable toxicity profile. The brief, easily managed myelosuppression observed did not impede repeated doses, further consolidating its feasibility as a therapeutic strategy.

The case report by Kawahara et al. added another layer to the growing body of evidence supporting the use of pulse cyclophosphamide in managing steroid-refractory hepatic GVHD, even for pediatric patients. A teenager with Grade III GVHD was successfully treated with pulse cyclophosphamide without any changes in chimerism or infection development, substantiating the efficacy and safety of pulse cyclophosphamide in treating this challenging condition (18).

In our case, similar to the treatment strategies outlined in these studies, pulse cyclophosphamide was used for treating steroid-refractory GVHD. Our patient’s clinical course parallels the positive outcomes demonstrated in these studies, suggesting that pulse cyclophosphamide might be a viable strategy for such complex cases. Notably, our case expands on the literature by demonstrating the efficacy of pulse cyclophosphamide in managing Grade IV GVHD, a population not included in the previously mentioned studies (17, 18).

### 3.4 Pulse cyclophosphamide: an affordable and feasible approach amidst therapeutic gaps

While medical advancements have led to the development of newer agents like ruxolitinib, this medication has shown limited efficacy in the patient’s treatment, reflecting a significant gap in our understanding of effective treatment strategies for this population.

In contrast to several newer FDA-approved treatment options for steroid-refractory cGVHD, pulse cyclophosphamide offers a distinct advantage in terms of cost effectiveness and simplicity of administration. Unlike ruxolitinib, ibrutinib, and belumosudil, which require ongoing treatment and can incur substantial annual costs—reaching up to $179,507 for ruxolitinib, $181,535 for ibrutinib, and $232,500 for belumosudil—pulse cyclophosphamide necessitates only a single dose (19–22). This single-dose regimen not only simplifies treatment but also significantly reduces the financial burden on both patients and healthcare systems.

## 4 Conclusion

In conclusion, managing steroid-refractory hepatic GVHD can be challenging, especially when patients are unresponsive to common treatments like steroids, tacrolimus, and ruxolitinib. However, the substantial and lasting improvement seen in our patient following a single pulse dose of cyclophosphamide offers a promising and cost-effective alternative. Not only did this approach swiftly resolve the hepatic insufficiency, but it also provided a durable response without any GVHD relapse. The feasibility of administering pulse cyclophosphamide in an outpatient setting further enhances its appeal.

This case, as the first report that Grade IV hepatic GVHD showed a fast-onset, complete, and durable response to pulse cyclophosphamide, invites further research into cyclophosphamide as a treatment for GVHD in patients unresponsive to conventional therapies. Its validation through clinical trials could represent a significant advancement in GVHD management, leading to improved patient outcomes and quality of life.

## Data availability statement

The original contributions presented in the study are included in the article/supplementary material. Further inquiries can be directed to the corresponding author.

## Ethics statement

Written informed consent was obtained from the individual(s) for the publication of any potentially identifiable images or data included in this article.

## Author contributions

YC: Writing – original draft, Writing – review & editing, Project administration, Supervision. AA: Resources, Supervision, Writing – review & editing, Project administration. EF: Writing – review & editing, Writing – original draft. RB: Writing – original draft, Writing – review & editing. TT: Writing – original draft, Writing – review & editing. OZ: Writing – original draft, Writing – review & editing. GY: Writing – review & editing, Investigation, Resources. AL: Investigation, Resources, Writing – review & editing. KW: Investigation, Resources, Writing – review & editing. ET: Investigation, Resources, Writing – review & editing. PC: Investigation, Resources, Writing – review & editing, Conceptualization, Supervision.
